# Supplementary material for: Inefficacy of mallard flight responses to approaching vehicles
Source: PeerJ. 2024 Sep 25;12:e18124. doi: 10.7717/peerj.18124 (PMC11438428; doi:10.7717/peerj.18124)
Supplement: Supplemental Information 1 — Image source: Microsoft Powerpoint. [file peerj-12-18124-s001.pdf]

Supplemental Figures

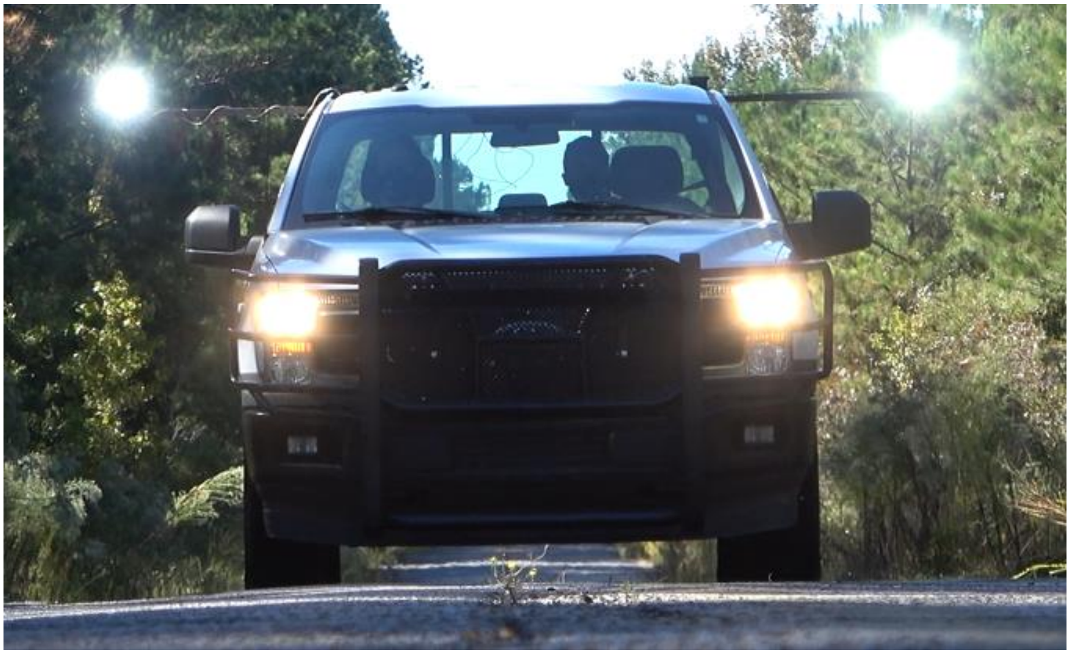

Figure S1. Experimental vehicle with lightbar attached.

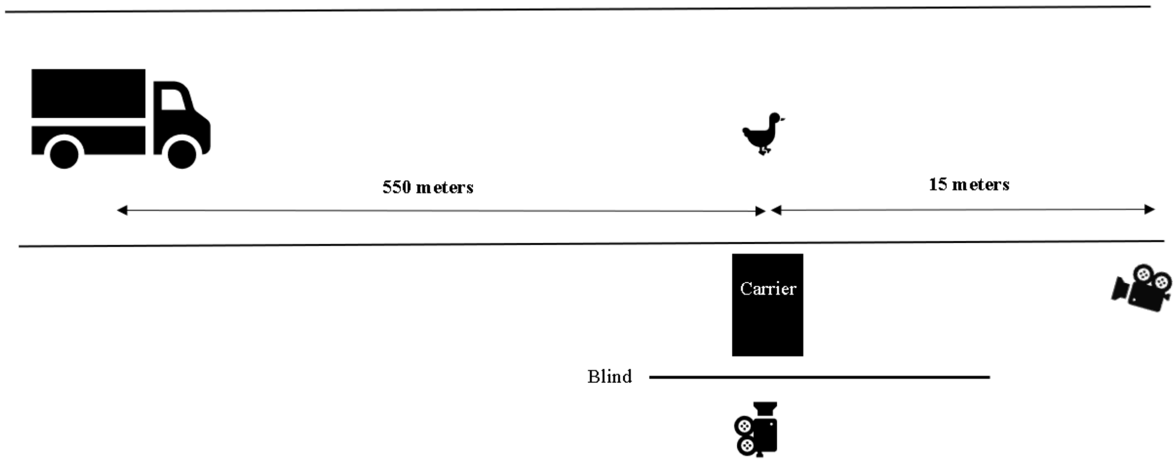

Figure S2. The location of the vehicle, mallard, and cameras at the beginning of an approach in the field.

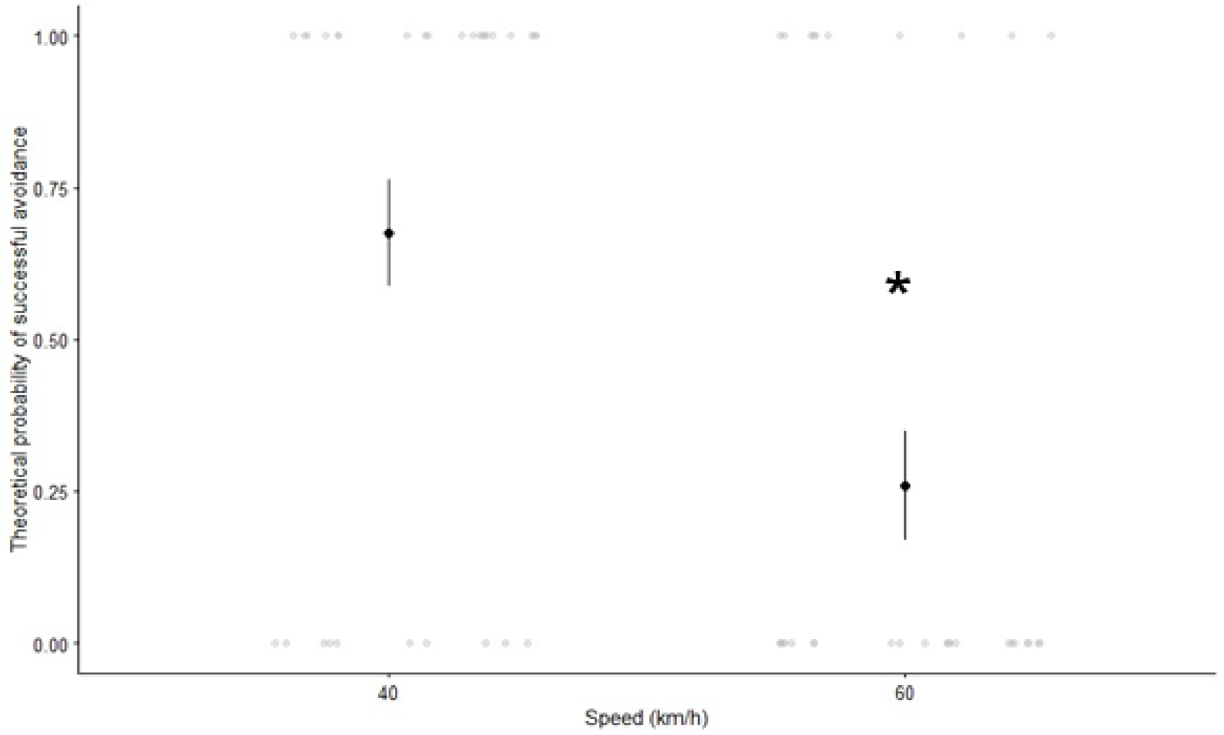

Figure S3. Probability of a mallard’s theoretical successful avoidance of the vehicle (TTC >1.0 s) at two experimental speeds during field vehicle approaches.
