# Supplementary material for: Inefficacy of mallard flight responses to approaching vehicles
Source: PeerJ. 2024 Sep 25;12:e18124. doi: 10.7717/peerj.18124 (PMC11438428; doi:10.7717/peerj.18124)
Supplement: Supplemental Information 2 [file peerj-12-18124-s002.pdf]

Arrive Checklist

1a. Lines 170 -171, Page 9 (simulated); Lines 223-224, Page 11 (field)

1b. See lines above – sampling units were individual animals.

2a. Animals per treatment: Lines 170-171, Page 9 (simulated); Lines 223-224, Page 11 (field)

Total animals: Lines 128-130, Page 7.

2b. Lines 130 – 132, Page 7.

3a. Animals were only excluded from analyses if they engaged in predetermined, unscorable behaviors (i.e., not acclimating in simulated trials, see lines 349-351, page 17; flying away before approach in field trials, see line lines 385 – 386, page 18). We also screened for outliers using a Grubbs test (Line 246, page 12), and presented results both with and without outliers.

3b. See lines above, also lines 366 – 371, pages 17-18 for discussion of outlier. See also lines 391 – 396, page 19 for discussion of inclusion/exclusion of mallards based on vehicle braking.

3c. Sample sizes for each analysis are included in results for each model (lines 358, 365, 375, 397, 407, 414, 420, 431)

4a. Mallards were run in the order caught from their holding area (Lines 171 – 172, page 9; 224-225, page 11).

4b. In simulated trials, treatments were cycled randomly (Line 171, page 9). In field trials, due to the temporal nature of Day/Night treatments, treatments were run in blocks with alternating speeds.

5. No particular strategy was undertaken to blind researchers to group allocation.

6a. Behavioral outcomes assessed are described in lines 238 – 246, page 12). Metrics of success are described in their respective experimental arena analysis (lines 277-289, page 14, simulated; lines 309-330, pages 15-16, field).

6b. Because this research was exploratory, no primary outcome was identified; see lines above for a discussion of outcomes of interest.

7a., 7b. Lines 246 – 254, page 12. If response variables were transformed to better meet assumptions of a model, this is noted in each model's section of Analysis methods (i.e., lines 274-276, page 13).

8a., 8b. Mallards are described in lines 122 – 129, page 6. All body masses are included in provided datasets.

9a., 9b. 9c., 9d. All procedures are described in methods section of this manuscript. Given that several experiments are contained in the methods section, identifying each line would result in highlighting nearly the entire section.

10a., 10b. Each of these metrics is listed in the results section accompanying their respective groups and model.

11. Abstract: Lines 13-27, page 2

12. Background: Choice of mallards as a model species is justified in lines 116 – 120, page 6.

13. Objectives: Lines 104-114, page 6.

14., 15., 16. See ethical note, lines 331-347, pages 16-17.

17. See Discussion section, beginning line 442, page 21.

18. See Conclusions section, beginning like 554, page 26.

19. Protocol was not registered prior to the study.

20. Data availability statement accompanies this manuscript. Data are attached as supplementary material, as well as available on Open Science Framework.

21. Declarations of interest and funding statement have been submitted along with manuscript.
